# The effects of two different dietary regimens during exercise on outcome of experimental acute kidney injury

**DOI:** 10.1186/s12950-022-00299-7

**Published:** 2022-03-02

**Authors:** Nazanin Sabet, Zahra Soltani, Mohammad Khaksari, Alireza Raji-Amirhasani

**Affiliations:** 1 Research Center of Tropical and Infectious Diseases, Kerman University of Medical Sciences, Kerman, Iran; 2 Endocrinology and Metabolism Research Center, Institute of Basic and Clinical Physiology Sciences, Faculty of Medicine, Kerman University of Medical Sciences, Kerman, Iran; 3 Department of Physiology and Pharmacology, Afzalipour Faculty of Medicine, Kerman University of Medical Sciences, Kerman, Iran; 4 Physiology Research Center, Institute of Neuropharmacology, Kerman University of Medical Sciences, Kerman, Iran

**Keywords:** Acute kidney injury, Exercise, SIRT1, Calorie restriction, Time restriction, Oxidative stress

## Abstract

**Background:**

Acute kidney injury (AKI) is a syndrome characterized by rapid loss of excretory function of kidney. Both exercise and some diets have been shown to increase silent information regulator (SIRT1) expression leading to reduction of kidney injury. In this study, the effect of two different diets during exercise on kidney function, oxidative stress, inflammation and also SIRT1 in AKI was investigated.

**Materials and methods:**

A number of rats were randomly divided into four groups; control without exercise, control with exercise, exercise + calorie restriction (CR), and exercise + time restriction (TR). Each group was divided into two subgroups of without AKI and with AKI (six rats in each group). Endurance exercise and diets were implemented before AKI. Serum urea and creatinine, urinary albumin, kidney malondialdehyde (MDA), total antioxidant capacity (TAC), transforming growth factor (TGF-β1), and SIRT1 levels, glomerular filtration rate (GFR) and relative kidney weight were measured before and 24 h after AKI induction.

**Results:**

After induction of kidney injury, serum urea and creatinine, urinary albumin, kidney MDA and TGF-β1 levels increased in rats with both previous exercise and no previous exercise, while GFR, and kidney TAC and SIRT1 levels significantly decreased. These changes after AKI were less in the group with previous exercise than in the group that had no exercise (*p* <0.001). The TR diet during exercise caused a less increase in serum urea (*p* <0.01) and creatinine (*p* <0.01), and urinary albumin (*p* <0.001) levels after the injury compared to the just exercise group. Also, both CR and TR diets during exercise caused less change in MDA (*p* <0.001) and TAC (*p* <0.05, *p* <0.001, respectively) levels compared to just exercise group.

**Conclusions:**

The results showed that exercise alone had no effect on preventing function impairment of kidney, oxidative stress, inflammation and also SIRT1 alteration following AKI, although these indexes were less among those with exercise than those without exercise. However, when the CR and TR diets were implemented during exercise, strong renoprotective effects appeared, and the protective effect of TR diet was greater.

## Background

Acute kidney injury (AKI) is a complex clinical disorder. It is also a syndrome of sudden loss of excretory function of kidney that is often associated with oliguria and mortality [1]. Despite technological advances that have been made in the treatment of kidney disease, AKI is still associated with poor clinical outcomes [2]. Causes of AKI can be pre-renal, intrinsic, and post-renal. Approximately 70% of AKI cases in the society are attributed to pre-renal causes. Decreased arterial blood pressure (for example due to heart failure or sepsis) leads to a decrease in glomerular filtration rate (GFR) [1, 3]. Patients with AKI eventually develop chronic kidney disease (CKD). They are also at risk for end-stage kidney disease (ESKD) and premature death [4].

Oxidative stress plays a crucial role in kidney injury [5]. Free radicals cause the lipid peroxidation, determined by overproduction of malondialdehyde (MDA), leading to cellular disruption in an organism [6]. When antioxidant defenses or total enzymatic and non-enzymatic antioxidant capacity (TAC) are weakened, body tissues become more prone to develop disease [7].

A number of inflammatory mediators are involved in the pathophysiology of kidney injury, including pro-inflammatory cytokines such as tumor necrosis factor (TNF-α), transforming growth factor (TGF-β1), interleukin-18 (IL-18), interleukin-1 (IL-1) and interleukin-6 (IL-6) [8]. TGF-β1 is a member of growth factor family that initiates a variety of pathophysiological processes at the onset of kidney injury, including apoptosis of tubular epithelial cell, lack of intrinsic cell differentiation and extracellular matrix deposition, which are associated with acute deterioration in renal function and fibrosis [9]. Some cells, such as macrophages, tubular epithelial cells and myofibroblasts, are able to secrete TGF-β at different stages of the renal fibrosis [10]. Multiple related factors such as oxidative stress, mitochondrial dysfunction, endoplasmic reticulum stress and a severe inflammatory response cause destruction of extracellular matrix, loss of kidney structure, impaired cellular homeostasis, and ultimately impaired renal function [10].

One of the molecules suggested as a target in the treatment of many diseases is the silent information regulator (SIRT1), a member of nicotinamide adenine dinucleotide-dependent histone deacetylase‎ that regulates various biological pathways by switching off the chromatin and suppressing transcription [11]. It is in relation to cellular energy metabolism, mitochondrial biogenesis, stress response, apoptosis, inflammation, and fibrosis [11, 12]. A collection of evidence suggests that SIRT1 plays a role in protecting cellular stress in kidney disease [13]. SIRT1 regulates the activity of several transcription factors that regulate renal cell homeostasis. SIRT1 expression decreases in patients with renal injury [14].

Physical activity and exercise promote health, help to maintain weight, and prevent health problems, vascular diseases and inflammatory diseases [15]. Regular muscle exercise has been shown to reduce oxidative stress and increase antioxidant enzymes such as superoxide dismutase (SOD), catalase and glutathione peroxidase (GPX) in various organs such as liver, kidney, heart and lungs [16].

Exercise may boost immune function in kidney disease and have anti-inflammatory effects [17]. It has been shown that regular and progressive aerobic exercise before AKI decreases the plasma creatinine and plasma levels, severity of tubular injury and caspase 3 levels within 48 h of reperfusion in Wistar rats [18]. Regular aerobic exercise and previous adaptation reduce morphological damage to the kidney in the form of interstitial edema in terms of mononuclear infiltration and loss of tubular brush border cells [19–21]. It has been reported that regular exercise before induction of diabetes improves metabolic control of renal function and decreased TGF-β expression in kidney tissue, which is associated with decreased fibronectin expression and a renoprotective effect [22]. It has also been reported that previous exercise or preconditioning can reduce damage to renal endothelial cell and improve angiogenesis [23].

It has been shown that diet manipulation, whether by changing calorie intake amount or food intake time, can lead to delayed onset and progression of disease and also a long and healthy life in most organisms [24]. Among dietary interventions, we can point to the classic calorie restriction diet (CR), in which daily caloric intake is normally reduced by 15 to 40% [25], and the time restriction diet (TR), in which daily food intake is limited to 4 to 12 h per day [26]. Neither of the diet has been proven to be superior to the other one. Diets that are associated with reduced energy intake exert their positive effects through weight loss, and reduced metabolism and oxidative damage [27].

CR diet has been shown to improve metabolic health and improve chronic metabolic diseases, such as type 2 diabetes and cardiovascular disorders. CR diet initiates a consistent defense mechanism that increases resistance to stress [28]. CR diet in particular has been shown to provide strong protection in laboratory models that induce ischemia-reperfusion (IR) injury in the brain, heart, liver and kidney [29]. Application of TR before renal injury has been shown to increase resistance to AKI and prevent the progression of interstitial fibrosis and oxidative stress [30].

Exercise, like CR and TR, affects health survival and disease recovery [23]. There is little evidence that CR along exercise can cause a greater increase in several health indicators [31]. Both exercise and CR have been shown to increase SIRT1 expression and ultimately reduce kidney injury [32, 33].

Athletes who are on the TR diet are able to maintain muscle mass, and reduce body fat and inflammatory markers. The TR diet, with 16 h of starvation and 8 h of nutrition, improved health-related biomarkers, reduced fat mass and maintained muscle mass in people who had endurance exercise [34]. This type of diet is used in athletes during the maintenance stages of training that aims to maintain muscle mass while reducing fat mass [34].

It is not clear whether the implementation of these diets in athletes affects their susceptibility towards diseases such as AKI or not. Therefore, in this study, the effects of CR and TR diets during exercise on the renal injury, oxidative stress and inflammation indexes, and SIRT1 in AKI male rats were investigated.

## Materials and methods

### Animals

In this study, male rats (aged 12-14 weeks and body weight of 200–250 g) were used. Animal care experiments were performed in accordance with standard ethical guidelines, and every effort was made to minimize animal suffering. All laboratory work was carried out according to the instructions of the Animal Care Committee of Kerman University of Medical Sciences (Ethics Code: IR.KMU.REC.1398.457). Male animals were placed in a cycle of 12 h of darkness and 12 h of light in the animal house of Kerman University of Medical Sciences at 22 to 23 °C, allowing them free access to food and water.

### Study groups

**Fig. 1:**
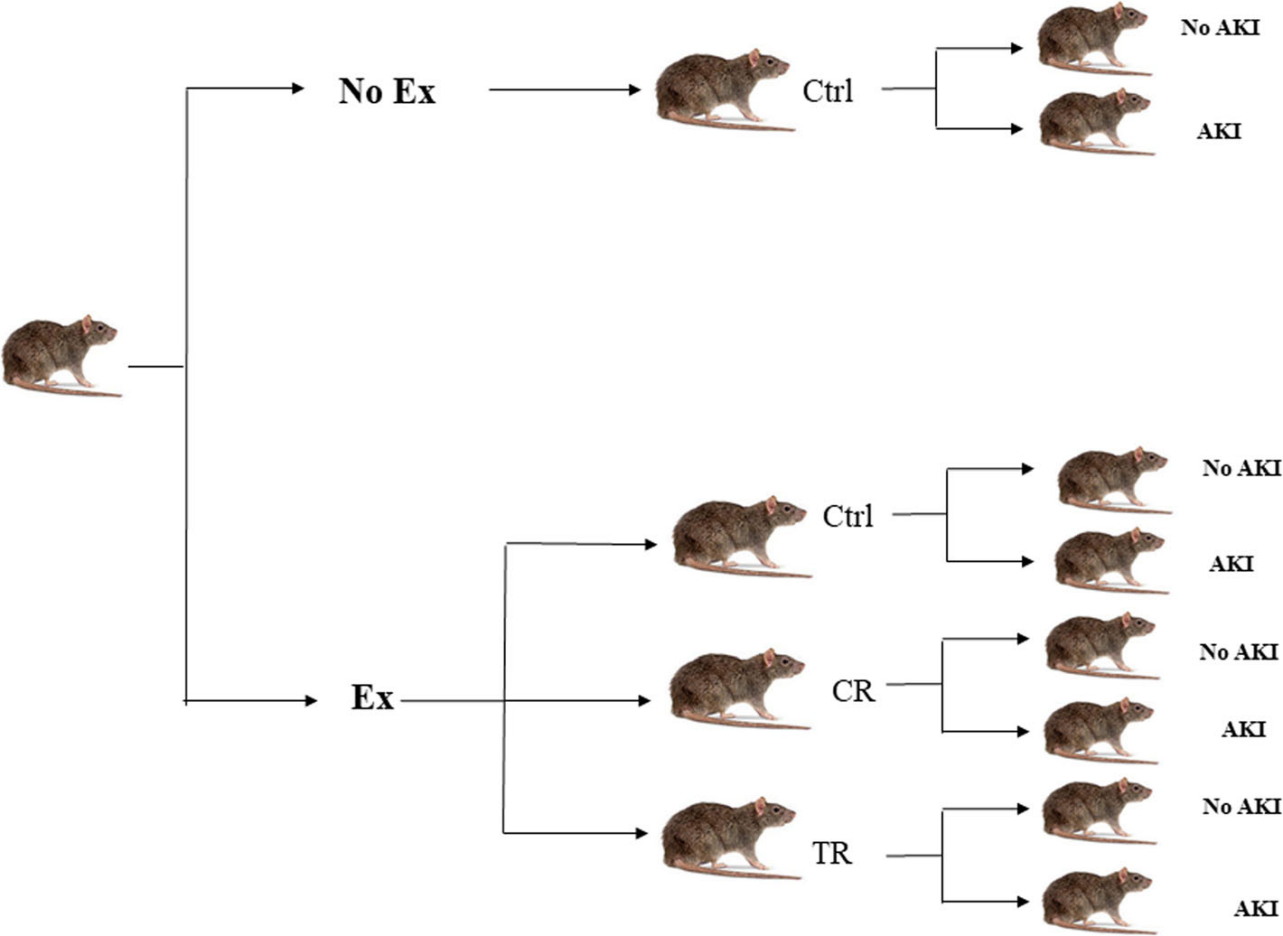
An overview of the study groups. AKI: Acute kidney injury; CR : Calorie restriction; Ctrl: Control; Ex: Exercise; TR: Time restriction

**Fig. 2:**
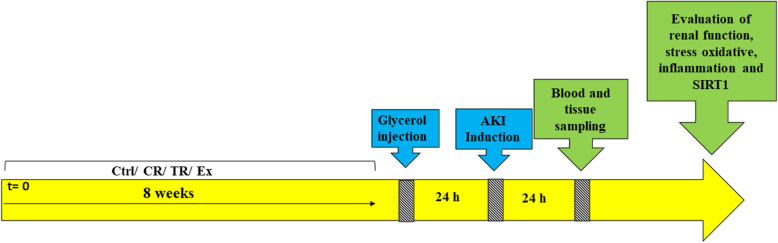
A schematic representation of the experimental protocol. AKI: Acute kidney injury; CR: Calorie restriction; Ctrl: Control; Ex: Exercise; SIRT1: Silent information regulator; TR: Time restriction

Two groups were used in the study to prove the induction of AKI, which included before and after AKI groups without exercise. The rest of the animals in the study had previous exercise, which were divided into three groups of without diet restriction (control), calorie restriction (CR) and time restriction (TR). Each one of these three groups also had two subgroups of before and after AKI (Fig. 1). The study protocol is also shown in Fig. 2.

### Study protocols

#### Implementation of CR and TR diets

To calculate the amount of food given to the CR group, first the amount of food consumed per week in the group that had free access to food was determined and using the average value, the amount of daily food consumption was calculated. Then 70% of the daily intake in the group that had free access to food was calculated and given to the CR group. This regimen was applied for 2 months before induction of AKI in the CR group [25]. In the TR group, five hours of free access to food was provided [26, 27].

#### Exercise protocol

Treadmill exercise was performed for eight weeks (5 days a week) until the day before AKI induction. For the first five days, the exercise was performed for 10 min a day at a speed of 20 m per minute on a flat surface. For the next 5 days, the duration of exercise increased by 10 min each day to 60 min at a speed of 26 m per minute without slope. For the rest of experiment time, the speed (26 m/min), duration (60 min), and 0° slope remained constant for eight weeks [35].

#### Induction of AKI

Two months after the start of the study, the animals in the AKI groups were deprived of water for 24 h and then injected with a single dose of 50% hypertonic glycerol solution (dissolved in saline). The injection was in a way that 10 ml/ kg of hypertonic glycerol solution was injected evenly into the muscles of both lower limbs [36]. With this method, nephropathy developed rapidly within 24 h of injection. Glycerol generally causes rhabdomyolysis, which eventually leads to myoglobinuria, ischemia, and nephrotoxicity in the kidney [37].

### Evaluation of kidney function indexes

#### Measurement of serum urea and creatinine, and urinary albumin

To measure serum urea and creatinine levels, one day before and one day after the induction of AKI, blood samples were collected from the ocular sinus and immediately centrifuged, and the serum was isolated. Then the measurements were performed using an analyzer (XL Selectra, Vital Scientific Company, Netherlands Country). Also, 24-hour urine of the animal was collected one day after the induction of AKI and the amount of albumin in the urine was measured using the aforementioned analyzer [25, 36].

#### Determining the GFR level

The GFR level was calculated using creatinine clearance according to the following formula [24].

GFR (ml/ min) = urinary creatinine concentration (mg/ dl) × urine volume in time unit (ml/ min).

plasma creatinine concentration (mg/ dl).

#### The ratio of kidney weight to body weight

The ratio of kidney weight to body weight is an indicator of hypertrophy. At the end of the study, the animals were killed under anesthesia and their kidneys were removed from the body and weighed. The weight of the animals was also determined before their death [38].

### Evaluation of inflammation and oxidative stress markers, and SIRT1 in kidney tissue

#### Measurement of MDA

The product of membrane lipid peroxidation, MDA, is considered as an oxidant that is measured by thiobarbituric acid (TBA) method. To measure MDA, a reaction mixture containing TBA, sodium dodecyl sulfate (SDS), 20% acetic acid (pH = 3.5) and distilled water were added to the homogenized tissue of kidney. The resulting mixture was heated at 90 °C for 45 min and after cooling in room temperature it was centrifuged at 10,000 g for 10 min to obtain a smooth solution. Then the adsorption of the supernatant was recorded at 532 nm. The amount of lipid peroxidation was expressed using the standard curve in nanomoles per milligram of protein [39].

#### Measurement of TAC

TAC was measured by ferric reducing antioxidant power (FRAP). For this purpose, homogenized samples of kidney tissue were first centrifuged at 10,000 g for 7 min. The supernatant was removed from the precipitate and diluted with distilled water for 5 times, and then quickly used to measure antioxidants. To measure the antioxidant activity, FRAP solution including sodium acetate, TPTZ (2, 4, 6-tri-pyridyl-s-3, 6 triazine) and ferric chloride were used. Then, from homogenized diluted samples, distilled water (as a control) and standard solution with predetermined dilutions (ferrous sulfate with concentrations of 125, 250, 500 and 1000 µM) were added to each test tube containing FRAP, and was heated at 37 °C in a hot water bath for 5 min. The absorbance of the samples was measured at wavelength of 593 nm, after removing the tubes from the hot water bath [39].

#### Measurement of TGF-β1 and SIRT1 levels

TGF-β1 and SIRT1 levels of kidney homogenized tissue were measured by ELISA method (Zellbio, Germany). In this case, the measurement was based on the reaction between the antigen and the antibody, and finally the adsorption was read at 450 nm. After placing the adsorption and concentration of standard solutions in Excel program, the standard curve was prepared. Concentration of the samples was determined based on the adsorption using the standard curve linear Eqs. [40–42].

### Statistical analysis

Two-way repeated measures ANOVA and T-test were used to compare quantitative variables between the study groups if the assumptions were observed (data normal distribution). A significant level of 0.05 was considered and statistical analyzes were performed by SPSS22 software.

## Results

### The effect of exercise and different diets on the kidney function indexes in AKI

**Fig. 3:**
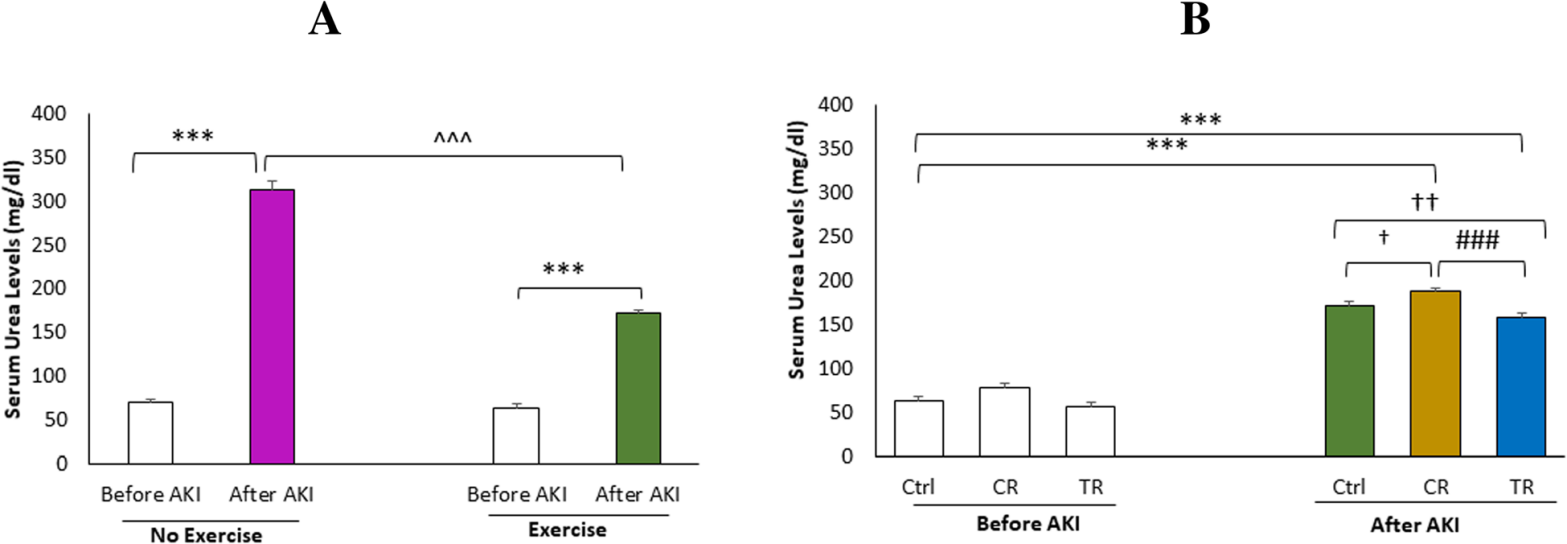
Serum urea levels (mg/dl) in the study groups (*n* = 6 in each group). Data are represented as mean ± SEM. **A** Serum urea levels in before and after AKI groups in non-exercised and exercised animals. ^***^p <0.001 vs. before AKI group in non-exercised or exercised rats. ^^^^^*p* <0.001 vs. after AKI group in non-exercised rats. **B** Serum urea levels in exercised groups. ^***^p <0.001 vs. Ctrl group before the injury. ^††^*p* <0.01 vs. Ctrl group after the injury. ^†^*p* <0.05 vs. Ctrl group after the injury. ^###^*p* <0.001 vs. CR group after the injury. AKI: Acute kidney injury, Ctrl: Control, CR: Caloric restriction: TR: Time restriction

**Fig. 4:**
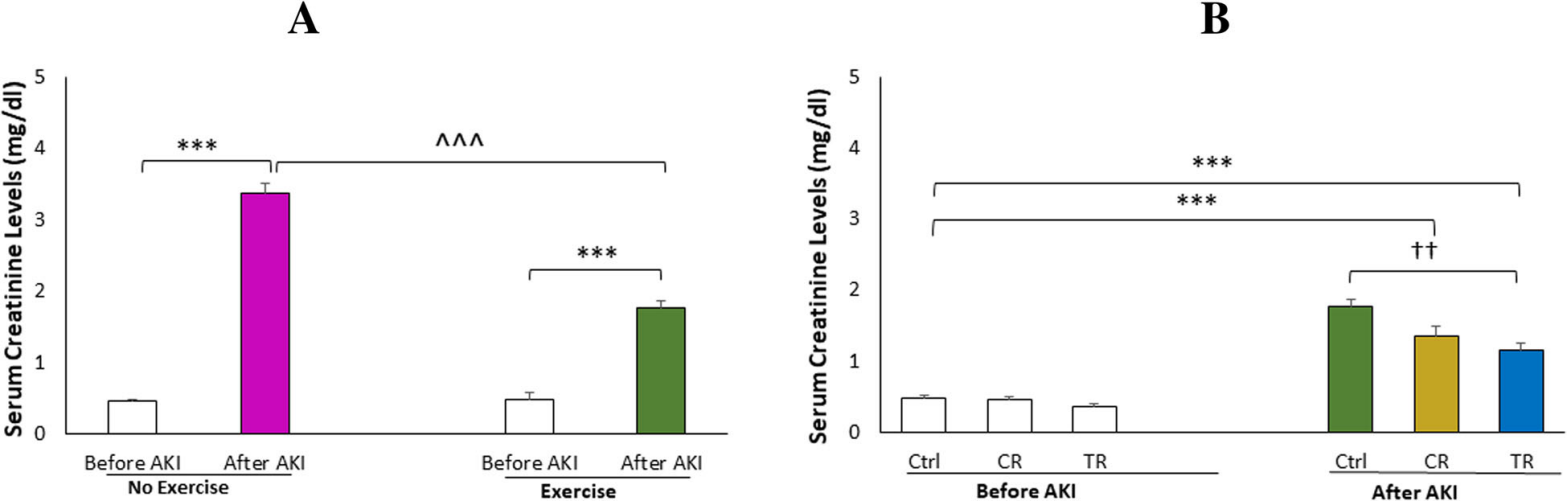
Serum creatinine levels (mg/dl) in the study groups (*n* = 6 in each group). Data are represented as mean ± SEM. **A** Serum creatinine levels in before and after AKI groups in non-exercised and exercised animals. ^***^p <0.001 vs. before AKI group in non-exercised or exercised rats. ^^^^^*p* <0.001 vs. after AKI group in non-exercised rats. **B** Serum creatinine levels in exercised groups. ^***^p <0.001 vs. Ctrl group before the injury. ^††^*p* <0.01 vs. Ctrl group after the injury. AKI: Acute kidney injury, Ctrl: Control, CR: Caloric restriction: TR: Time restriction

Serum urea and creatinine levels increased after AKI, both in the group with previous exercise and in the group without previous exercise compared to before AKI (*p *<0.001) (Figs. 3A, 4A). However, this increase in the exercised group was less than the group that had no exercise (*p* <0.001) (Fig. 3A). In the exercised groups, the increase in urea after the injury in the TR group was less than the Ctrl and CR groups (*p* <0.01 and *p* <0.001, respectively) (Fig. 3B) and in the CR group was more than the Ctrl group (*p* <0.05) (Fig. 3B). The increase in creatinine levels was lower in the TR group compared to the Ctrl group (*p* <0.01) (Fig. 4B). The levels of urea and creatinine after AKI in the TR and CR groups were higher than that before AKI (*p* <0.001). Also, the amount of urea and creatinine after AKI was higher in the TR and CR groups compared to Ctrl group before the injury (*p* <0.001) (Figs. 3B and 4B).

**Fig. 5:**
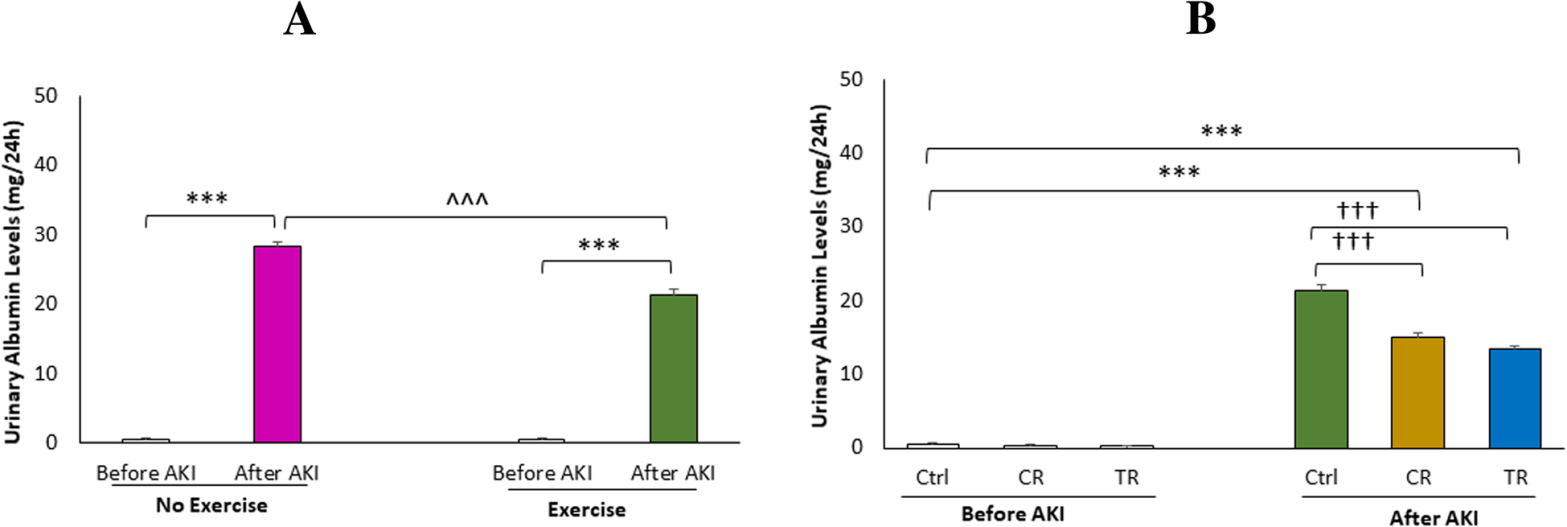
Urinary albumin levels (mg/24 h) in the study groups (*n* = 6 in each group). Data are represented as mean ± SEM. **A** Urinary albumin levels in before and after AKI groups in non-exercised and exercised animals. ^***^p <0.001 vs. before AKI group in non-exercised or exercised rats. ^^^^^*p* <0.001 vs. after AKI group in non-exercised rats. **B** Urinary albumin levels in exercised groups. ^***^p <0.001 vs. Ctrl group before the injury. ^†††^*p* <0.001 vs. Ctrl group after the injury. AKI: Acute kidney injury, Ctrl: Control, CR: Caloric restriction: TR: Time restriction

The levels of urinary albumin increased after the injury in both exercised and non-exercised groups compared to before the injury (*p* <0.001) (Fig. 5A). This increase in the exercised group was less than the group that had no exercise (*p* <0.001) (Fig. 5A). In the exercised groups, urinary albumin levels after the injury in CR and TR groups were less than that Ctrl group (*p* <0.001) (Fig. 5B). The levels of urinary albumin after AKI in the TR and CR groups were higher than that before AKI (*p* <0.001). Albumin levels after AKI in CR and TR groups also were higher than that Ctrl group before the injury (*p* <0.001) (Fig. 5B).

**Fig. 6:**
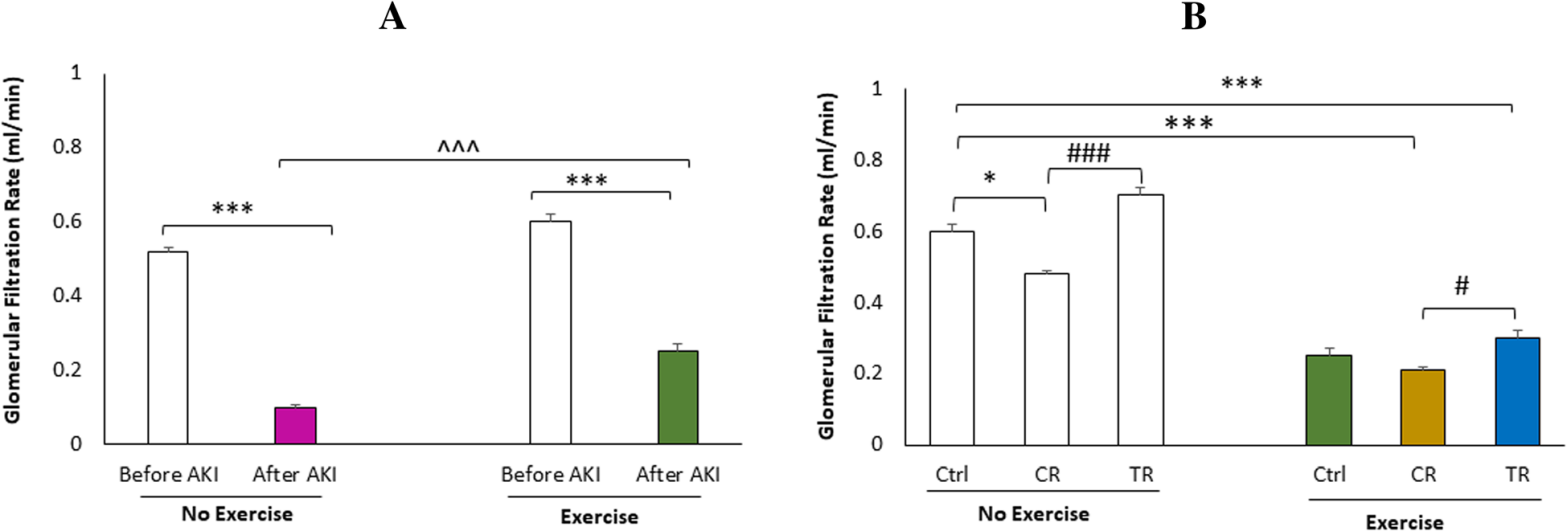
Glomerular filtration rate (GFR) (ml/min) levels in the study groups (*n* = 6 in each group). Data are represented as mean ± SEM. **A** GFR levels in before and after AKI groups in non-exercised and exercised animals. ^***^p <0.001 vs. before AKI group in non-exercised or exercised rats. ^^^^^*p* <0.001 vs. after AKI group in non-exercised rats. **B** GFR levels in exercised groups. ^***^p <0.001 vs. Ctrl group before the injury. ^*^p <0.05 vs. Ctrl group before the injury. ^###^*p* <0.001 vs. CR group before the injury. ^#^*p *<0.05 vs. CR group after the injury. AKI: Acute kidney injury, Ctrl: Control, CR: Caloric restriction: TR: Time restriction

Both in the group with previous exercise and in the group without previous exercise, the GFR decreased after AKI compared to before AKI (*p* <0.001) (Fig. 6A). However, this decrease in the exercised group was less than the group that had no exercise (*p* <0.001) (Fig. 6A). In the exercise condition, this rate before AKI was lower in CR group compared to Ctrl and TR groups (*p* <0.05 and *p* <0.001, respectively) (Fig. 6B). The decrease in GFR level after the injury in TR group was less than CR group (*P* <0.05) (Fig. 6B). The levels of GFR after AKI in the TR and CR groups were less than that before AKI (*p* <0.001). GFR levels after the injury in CR and TR groups were appeared lower than that Ctrl group before the injury (*p* <0.001) (Fig. 6B).

**Fig. 7:**
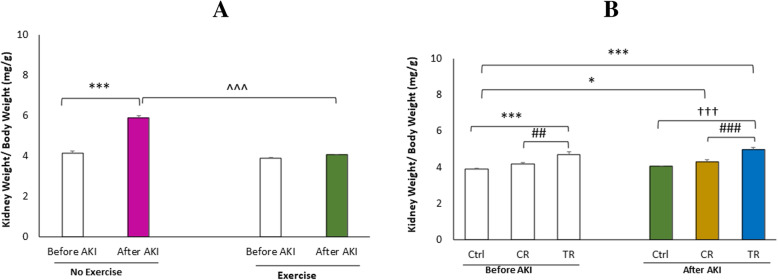
Kidney Weight/ Body Weight ratio (mg/g) in the study groups (*n* = 6 in each group). Data are represented as mean ± SEM. **A** Kidney Weight/ Body Weight ratio in before and after AKI groups in non-exercised and exercised animals. *** *p* <0.001 vs. before AKI group in the non-exercised rats. ^^^^^*p* <0.001 vs. after AKI group in non- exercised rats. **B** Kidney Weight/ Body Weight ratio in exercised groups. ^***^p <0.001 vs. Ctrl group before the injury. ^*^p <0.05 vs. Ctrl group before the injury. ^###^*p* <0.001 vs. CR group after the injury. ^##^*p* <0.01 vs. CR group before the injury. ^†††^*p* <0.01 vs. Ctrl group after the injury. AKI: Acute kidney injury, Ctrl: Control, CR: Caloric restriction: TR: Time restriction

The ratio of kidney weight to body weight after the injury only in the group without previous exercise increased compared to before the injury (*p* <0.001) (Fig. 7A). In the exercise condition, the ratio of kidney weight to body weight before AKI was higher in the TR group than the Ctrl ang CR groups (*p* <0.001 and *p* <0.01, respectively) (Fig. 7B). Also, the ratio after the injury was shown higher in the TR group than the Ctrl and CR groups (*p* <0.001) (Fig. 7B). This ratio in CR and TR groups after the injury showed no difference compared to before the injury (*p*= 0.51 and *p*= 0.17, respectively). The ratio in the CR and TR groups after the injury was higher than the Ctrl group before the injury (*p* <0.05 and *p* <0.001, respectively) (Fig. 7B).

### The effect of exercise and different diets on the levels of inflammation and oxidative stress markers, and SIRT1 in the kidney following AKI

#### MDA levels in the study groups

**Fig. 8:**
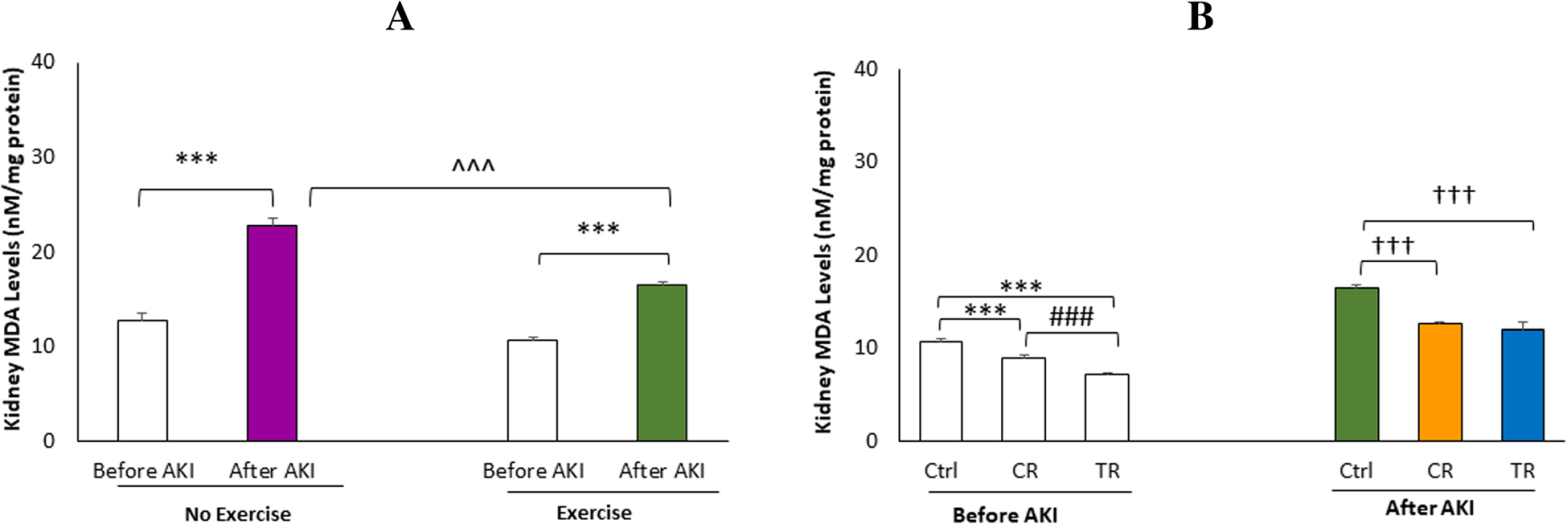
Kidney malondialdehyde (MDA) levels (nM/mg protein) in the study groups (*n* = 6 in each group). Data are represented as mean ± SEM. **A** Kidney MDA levels in before and after AKI groups in non-exercised and exercised animals. ^***^p <0.001 vs. before AKI group in non-exercised or exercised rats. ^^^^^*p* <0.001 vs. after AKI group in non-exercised rats. **B** Kidney MDA levels in exercised groups. ^***^p <0.001 vs. Ctrl group before the injury. ^###^*p* <0.001 vs. CR group before the injury. ^†††^*p* <0.001 vs. Ctrl group after the injury. AKI: Acute kidney injury, Ctrl: Control, CR: Caloric restriction: TR: Time restriction

Kidney tissue MDA levels in the study groups are shown in Fig. 8. An increase in MDA levels after the injury compared to before the injury appeared in both exercised and non-exercised groups (*p* <0.001) (Fig. 8A). However, this increase in the exercised group was less than the group that had no exercise (*P*<0.001) (Fig. 8A). In the exercise condition, the level of MDA before AKI in the CR and TR group was lower than the Ctrl group (*p* <0.001) (Fig. 8B) and this increase in TR group was lower than CR group (*p* <0.001) (Fig. 8B). The MDA levels in the CR and TR groups after AKI were lower than the Ctrl group (*p* <0.001) (Fig. 8B). The levels of MDA after AKI in the TR and CR groups were observed higher than that before AKI (*p* <0.001). The MDA levels in CR and TR groups after the injury showed no difference compared to Ctrl group before the injury (*p*= 0.06 and *p*= 0.26, respectively) (Fig. 8B).

#### TAC levels in the study groups

**Fig. 9:**
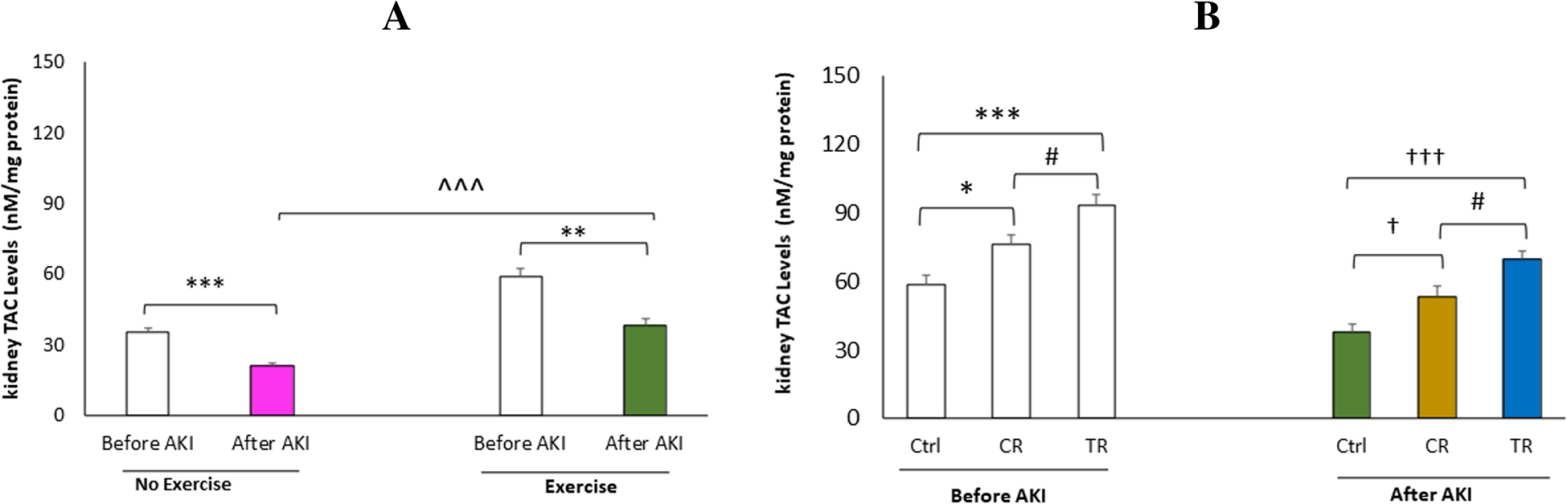
Kidney total antioxidant capacity (TAC) (nM/mg protein) levels in the study groups (*n* = 6 in each group). Information is represented as mean ± SEM. **A** Kidney TAC levels in before and after AKI groups in non-exercised and exercised animals. ^***^p <0.001 vs. before AKI group in non-exercised rats. ^**^p <0.01 vs. before AKI group in exercised rats. ^^^^^*p* <0.001 vs. after AKI group in non-exercised rats. **B** Kidney TAC levels in exercised groups. ^***^p <0.001 vs. Ctrl group before AKI. ^*^p <0.05 vs. Ctrl group before AKI. ^#^*p* <0.05 vs. CR group before or after AKI. ^†††^* p* <0.001 vs. Ctrl group after AKI. ^†^*p *<0.05 vs. Ctrl group after AKI. AKI: Acute kidney injury, Ctrl: Control, CR: Caloric restriction: TR: Time restriction

Figure 9 shows the levels of kidney tissue TAC in the study groups. After AKI, the levels of TAC decreased in the non-exercised and exercised groups compared to before AKI (*p* <0.001 and *p* <0.01, respectively) (Fig. 9A). This decrease in the exercised group was less than the group that had no exercise (*p* <0.001) (Fig. 9A). In the exercise condition, the levels of TAC before AKI in CR and TR groups were more than Ctrl group (*p* <0.05 and *p* <0.001, respectively) (Fig. 9B) and this increase in TR group was higher than CR group (*p* <0.05) (Fig. 9B). The decrease in TAC levels after AKI was less in CR and TR groups than in Ctrl group (*p* <0.05 and *p* <0.001, respectively) (Fig. 9B) and this decrease was appeared less in TR group compared to CR group (*p *<0.05) (Fig. 9B). The levels of TAC after AKI were lower in the TR and CR groups than that before AKI (*p* <0.001). Also, no difference was observed in the TAC levels of CR and TR groups after the injury compared to Ctrl group before the injury (*p*= 0.59 and *p*= 0.16, respectively) (Fig. 9B).

#### TGF-β1 levels in the study groups

**Fig. 10:**
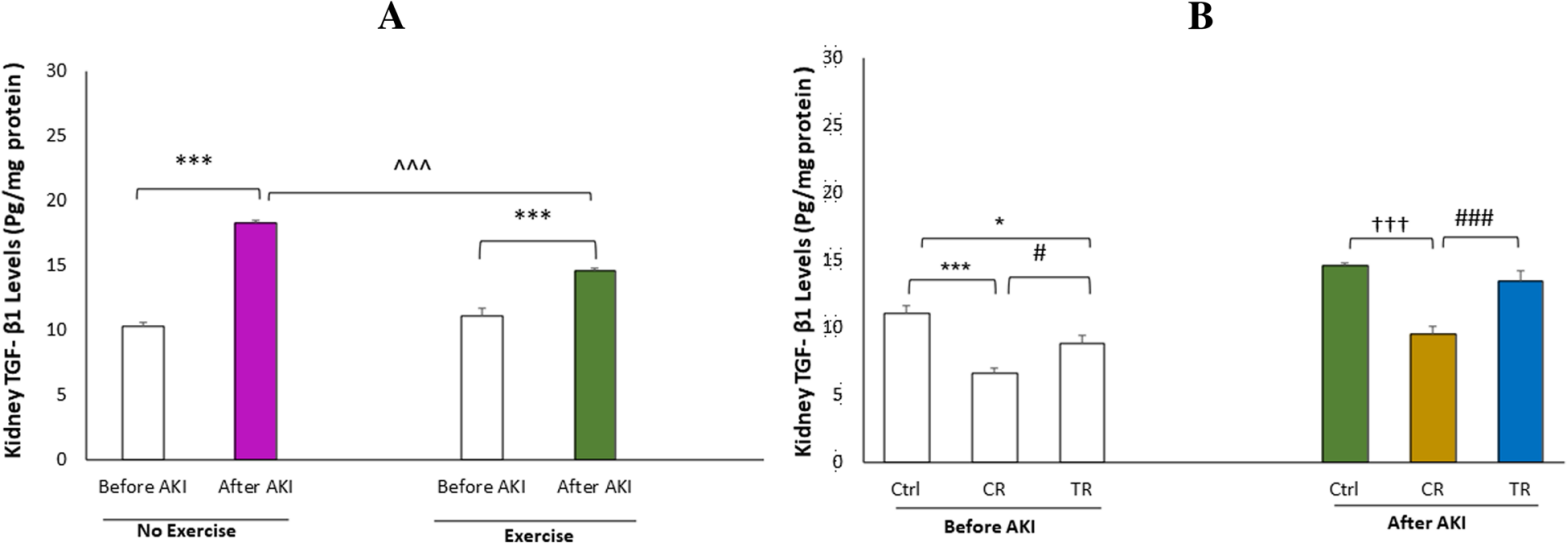
Kidney transforming growth factor (TGF-β1) (pg/mg protein) in the study groups (*n* = 6 in each group). Data are represented as mean ± SEM. **A** Kidney TGF-β1 levels in before and after AKI groups in non-exercised and exercised animals. ^***^p <0.001 vs. before AKI group in non-exercised or exercised rats. ^^^^^*p* <0.001 vs. after AKI group in non-exercised rats. **B** Kidney TGF-β1 levels in exercised groups. ^***^p <0.001 vs. Ctrl group before AKI. ^*^p <0.05 vs. Ctrl group before AKI. ^###^*p* <0.001 vs. CR group after the injury. ^#^*p* <0.05 vs. CR group before the injury. ^†††^*p* <0.001 vs. Ctrl group after the injury. AKI: Acute kidney injury, Ctrl: Control, CR: Caloric restriction: TR: Time restriction

Kidney tissue TGF-β levels were measured in the study groups (Fig. 10). In both exercised and non-exercised groups, the levels of TGF-β1 increased after the injury compared to before the injury (*p* <0.001) (Fig. 10A). This increase in the exercised group was less than the non-exercised group (*p* <0.001) (Fig. 10A). In the exercise condition, the levels of TGF-β1 before AKI in CR and TR groups were lower than Ctrl group (*p* <0.001 and *p* <0.05, respectively) (Fig. 10B) and in CR group was lower than TR group (*p* <0.05) (Fig. 10B). Also, in the exercise condition, the levels of TGF-β1 after the injury increased less in CR group than Ctrl and TR groups (*p* <0.001) (Fig. 10B). The levels after AKI in the TR and CR groups were higher than that before AKI (*p* <0.001). No difference in TGF-β1 levels was observed after the injury in CR and TR groups compared to Ctrl group before the injury (*p*= 0.06 and *p*= 0.27, respectively) (Fig. 10B).

#### SIRT1 levels in the study groups

**Fig. 11:**
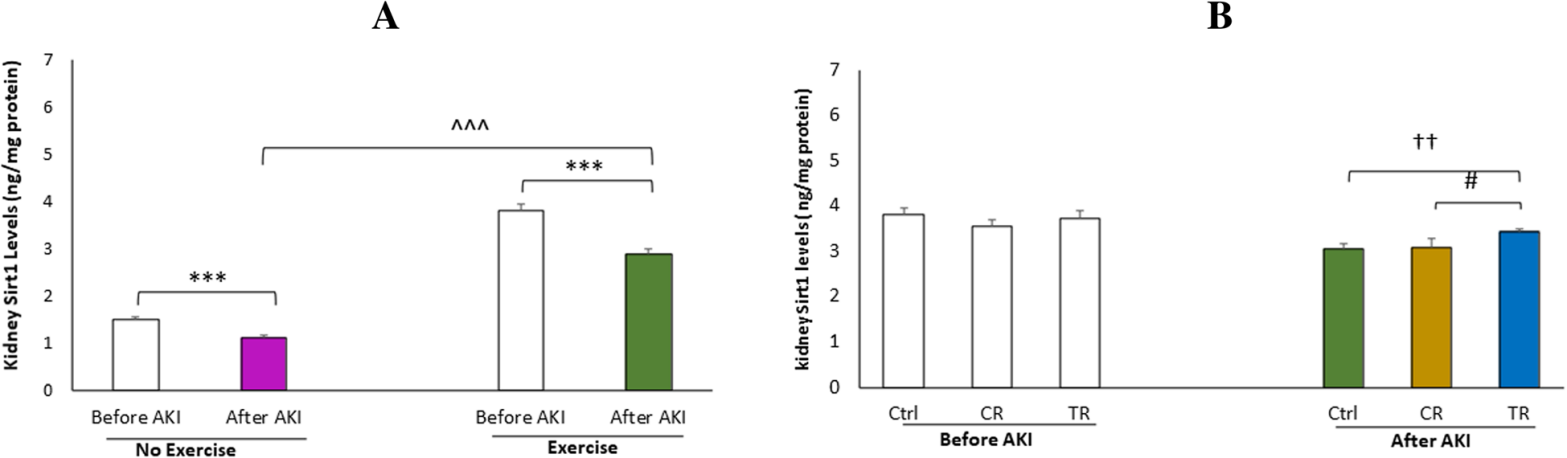
Kidney silent information regulator (SIRT1) (ng/mg protein) levels in the study groups (*n* = 6 in each group). Data are expressed as mean ± SEM. **A** Kidney SIRT1 levels in before and after AKI groups in non-exercised and exercised animals. ^***^p <0.001 vs. before AKI group in non-exercised or exercised rats. ^^^^^*p* <0.001 vs. after AKI group in non-exercised rats. **B** Kidney SIRT1 levels in exercised groups. ^††^*p* <0.01 vs. Ctrl group after AKI. ^#^*p* <0.05 vs. CR group after the injury. AKI: Acute kidney injury, Ctrl: Control, CR: Caloric restriction, TR: Time restriction

Kidney tissue SIRT1 levels in the study groups are shown in Fig. 11. The level of SIRT1 decreased after the injury compared to before the injury (*p* <0.001) (Fig. 11A). This decrease in the exercised group was less than the non-exercised group (*p* <0.001) (Fig. 11A). After the injury, in the exercise condition, the levels of SIRT1 in exercised group were higher in the TR group compared to the Ctrl and CR groups (*p* <0.01 and *p* <0.05, respectively) (Fig. 11B). The levels after AKI in the CR and TR groups were observed lower than that before AKI (*p* <0.01 and *p* <0.05, respectively) (Fig. 11B). No difference was observed in the SIRT1 levels of CR and TR groups after the injury compared to Ctrl group before the injury (*p*= 0.29 and *p*= 0.06, respectively) (Fig. 11B).

## Discussion

In this study, for the first time, the effect of two types of dietary regimen during exercise on AKI outcomes was investigated. Following eight weeks of moderate-intensity endurance exercise along the use of different diets, the following results were obtained:

(1) Following AKI, the serum urea and creatinine, and urinary albumin levels increased in the exercised and non-exercised groups, and this increase was less in the exercised rats than in non-exercised ones. Serum urea levels were lower in the exercised group with TR than the exercised group with CR and just exercised group, while urinary albumin was lower in exercised groups with CR and TR diets than just exercised groups. (2) GFR levels decreased after AKI in exercised and non-exercised groups, and this decrease was less in the exercised rats than non-exercised ones. It was also less in the exercised rats on TR diet than in exercised rats on CR diet. (3) The relative weight of the kidney increased after the injury only in the non-exercised group. (4) The MDA levels increased and TAC levels decreased following the injury in the exercised and non-exercised groups. These changes were less in the exercised groups than the non-exercised groups and also less in exercised rats with prescribed regimens than rats that only performed exercise. Also, the level of TAC reduction was lower in the exercised group with TR than in exercised group with CR. (5) Increase in inflammatory factor (TGF-β1) levels following the injury in exercised and non-exercised groups showed that the increase was less in the exercised group than the non-exercised group and also, it was less in the exercised group with CR compared to just exercise group and exercised group with TR. (6) Decrease in SIRT1 levels following AKI in exercised and non-exercised groups revealed that the decrease in exercised group was less than the non-exercised group. This decrease was also less in the exercised group with TR after the injury than in just exercised group.

Rhabdomyolysis, the leading cause of AKI, is common in athletes and can occur 1 to 10 h after exercise and resolve after 2 to 10 days [43]. However, the effectiveness of exercise as a treatment depends on its duration, intensity and type [44]. In this study, the effect of TR and CR diets during exercise was investigated. The beneficial effects of these diets depend on many factors including age, physical activity and disease status [45]. The TR diet is a form of Islamic fasting [46]. A CR diet however, in which daily calorie intake is limited, can also be an intervention for athletes who want to control their body weight and increase their physical function and energy [31]. CR in athletes reduces the risk of metabolic disease and mortality [45].

Increased serum urea and creatinine levels following AKI in the present study have also been shown in other AKI models, such as the use of cisplatin [47]. Lower increase in the serum urea and creatinine levels in the exercised group compared to non-exercised group has been confirmed in the study of Weslei et al. (2019) [18]. De lima et al. (2019) showed that regular exercise with moderate intensity for four weeks before AKI causes a decrease in serum urea and creatinine levels, and tubular injury [18]. In this study, it was shown that TR diet combined with exercise resulted in a lower increase in serum urea and creatinine levels following AKI. Prevention of excessive increase in serum urea and creatinine levels following AKI by the use of TR diet has also been reported [30], which its mechanism of action is still unknown. Therefore, exercise prevents excessive increase in serum urea and creatinine levels after AKI, and when combined with TR diet, its effect is greater. A study showed that TR diet in athletes did not change the renal function indexes including serum urea and creatinine levels [48]. The difference in the results could be due to differences in the studied species, and intensity and duration of exercise.

The increase in urinary albumin levels after AKI in the present study has also been confirmed in the study of Palm et al. (2004) [49]. One possible cause of the increase in urinary albumin is increased kidney injury and tubular damage [50]. Consistent with our result that revealed less increase in urinary albumin after AKI in the group with previous exercise, it has been shown that regular exercise for 10 weeks reduces urinary albumin levels in diabetic rats [51]. Also, endurance exercise with moderate intensity for four weeks before induction of diabetes decreases urinary albumin levels after the injury [52]. There are also contradictory results regarding the effects of exercise on proteinuria, and evidence suggests that the intensity of exercise is very important in this area [53–55]. Some studies have shown that high-intensity exercise increases albuminuria in laboratory animals and humans, while moderate-intensity and regular exercise prevents albuminuria / proteinuria in STZ-induced diabetic rats [53–55]. Although exercise prevents increase in urinary albumin levels following AKI, when combined with TR and CR diets, its effect is greater. According to the searching that we carried out, no study has been conducted to investigate the effects of these two diets on the reduction of proteinuria to this date.

In the present study, similar to another study [56], GFR decreased following AKI, and this decrease was less in exercised group compared to non-exercised group. It has been shown that performing eight weeks of moderate-intensity endurance exercise before induction of diabetes improves GFR after the injury and prevents a large decrease in GFR [54]. Toyama et al. (2010) reported that exercise for 12 weeks improves GFR in patients with CKD [55]. Although the mechanism by which moderate-intensity exercise improves renal function is not well understood, there is evidence to suggest that better metabolic control, reduced oxidative stress, and increased nitric oxide (NO) production may play a role in this protective process [51, 52]. Decreased GFR before the injury was observed in the exercised rats on CR diet in the present study. In one study, the CR diet alone reduced GFR by reducing tubular hypertrophy [56]. It is possible that the reduction of tubular hypertrophy in the CR diet group caused the decrease in GFR was not compensated in the CR diet group with exercise. The decrease in GFR in the TR diet group during exercise was less than the CR diet group during exercise, which is probably due to compensatory tubular hypertrophy both before and after the injury [57].

Amaral et al., similar to the present study, reported that the relative weight of kidney increased after kidney injury, and eight weeks of moderate-intensity endurance exercise before induction of diabetes prevented the increase in relative weight of the kidney after the injury [22]. The reason for the increase in relative kidney weight in the TR diet group during exercise, before and after AKI in this study, may be due to the increase in compensatory hypertrophy, which is in contrast with the CR diet group during exercise. In a study, CR diet prevented a relative increase in heart weight associated with aging [58]. Therefore, CR with exercise has a more effect on preventing the increase in compensatory renal hypertrophy after AKI.

Therefore, it can be concluded that in the present study, exercise probably decreased renal injury by reducing oxidative stress [55], which is the cause of tubular damage, and by increasing renal NO [54] and proliferation of kidney tissue cells [59] prevented GFR reduction. Also, compensatory hypertrophy in TR group led to less reduction in GFR, increased relative kidney weight, and improved renal function, which could be observed by smaller increase in serum urea and creatinine, and urinary albumin levels. In the TR diet group with exercise, these effects were reinforced and played a more effective role in improving kidney function.

The increase in lipid peroxidation and the decrease in antioxidant defense after AKI in this study are also confirmed by Ibrahim et al. (2008), [60]. Evidence suggests that exercise before AKI prevents the subsequent increase in oxidative stress [58, 60]. Exercise is said to strike a balance between the oxidant and antioxidant systems [61]. The findings of Húngaro et al. (2020) study are contradictory to the results of our study, as they reported that moderate-intensity endurance exercise for four weeks before AKI could not prevent oxidative stress [62]. The reason for the difference between this study and our study could be the difference in the duration of exercise and the type of animal used in these studies. Walsh et al. (2014), similar to the results of present study, reported that dietary restriction prevents the progression of oxidative stress after AKI [63]. The protective mechanism of this regimen is still unknown, but it has been shown that this protective effect is probably exerted by an increase in antioxidant factors [64] that prevent DNA oxidative damage induced by kidney injury [64]. One study even suggested that a reduction in oxidative stress by CR diet may be due to weight loss [65]. Another study found that exercise did not prevent oxidative stress in brain tissue, but the CR diet prevented the production of ROS products and established a balance between oxidant and antioxidant systems [66]. The difference could be due to differences in the type, intensity and duration of exercise, as well as the studied tissue and the study conditions. Wycherley et al. (2008) showed that CR with exercise reduced serum MDA, possibly due to weight loss [67]. Reduction of oxidative stress may also be due to reduced insulin resistance and blood pressure following CR diet [67].

Although in this study weight gain was observed during exercise with TR diet, kidney weight loss could have been the cause of reduced oxidative stress in the CR diet. TR has been shown to prevent mitochondrial fragmentation associated with severe mitochondrial dysfunction, overproduction of free radicals, and worsening of AKI [64, 68, 69]. At present, the molecular mechanisms of this effect are unclear, but it is possible that the nuclear factor-erythroid factor 2-related factor 2 (NRF2) and peroxisome proliferator-activated receptor-γ coactivator 1-α (PGC1α), are both oxidation-sensitive transcriptional regulators, which are activated by nutrient restriction, affect mitochondrial homeostasis and play a role in the beneficial effects of TR [70–72]. It seems that the reduction of oxidative stress both before and after AKI in the TR group during exercise may be due to the effect of TR on preventing mitochondrial damage following regulating oxidation-sensitive transcriptional factors, which requires further research.

In the present study, similar to another study, AKI increased the level of TGF-β1 in kidney tissue [73]. It has been shown that regular moderate-intensity exercise before induction of diabetes partially reduces the progression of renal fibrosis by significantly reducing advanced glycation end products (AGE), which consequently reduces the production of TGF-β1 in mesangial, fibroblasts and tubular cells [74]. The results of Húngaro et al. (2020) study contradict the findings of present study, as they reported that moderate-intensity endurance exercise for four weeks before AKI could not prevent inflammation [62]. A reason for the increased expression of TGF-β1 is the increased expression of fibronectin and type four collagen following kidney damage, which in turn causes the accumulation of extracellular matrix and the progression of fibrosis [75]. Exercise before induction of kidney injury, reduces the expression of fibronectin and type four collagen in kidney tissue and prevents the progression of fibrosis by reducing TGF-β1 production after AKI [22]. These effects of exercise in the present study might have caused the level of TGF-β1 after AKI in the exercised group to be less than in the non-exercised group.

In other studies, similar to the present study, it has been reported that dietary restriction prevents the progression of renal fibrosis by reducing TGF-β production after AKI [30, 76]. Liu et al. (2020) found that CR diet reduces TGF-β1 and ultimately fibrosis in aging-related kidney disorders [77]. The CR-induced protection against fibrosis could be due to reduced oxidative stress, which reduces mitogen-activated protein kinase (MAPK) activity, activator protein-1 (AP-1) regulation, and TGFβ1 expression and signaling [78]. Given the effect of CR diet during exercise on oxidative stress in the present study, it is likely that CR during exercise reduces TGFβ1 by reducing oxidative stress.

A decrease in SIRT1 levels in kidney tissue following AKI was observed in this study similar to the study of Zhong et al. (2018), [32]. Decreased SIRT1 expression is associated with increased nuclear factor kappa B (NF-κB) acetylation [79]. Exercise suppresses NF- κB activity and inflammation by increasing SIRT1 expression in the kidney [33]. In addition, exercise induces mitochondrial complex expression and the release of antioxidant enzymes by improving the activity of SIRT1 enzyme in the kidney [80]. Prevention of SIRT1 reduction after AKI by previous exercise may have contributed to the reduction of oxidative stress and TGFβ1 after AKI in the exercised group. Marton and his colleagues showed that in metabolic disorders caused by aging, exercise could not prevent the reduction of SIRT1 in the cerebellum [81], which is contrary to our results. This difference could be due to the age of the animal, duration of exercise and the type of tissue studied. In the present study, an increase in SIRT1 after AKI was observed in the TR group during exercise. In another study, the TR diet restored the circadian rhythm of SIRT1 expression in the liver following metabolic disorders [82].

Therefore, in AKI, due to kidney injury, there is an increase in oxidative stress and inflammation, and also a decrease in renal function indexes and SIRT1 levels, which are less in the presence of exercise. However, with the application of CR and TR diets, especially TR, these changes are reduced in the presence of exercise. Applying the TR regimen before kidney injury prevents structural damage to the kidney and renal function reduction in kidney injury [83]. These effects are associated with the response to prevent proliferation of epithelial cells of damaged tubules and suppression of extracellular signal-regulated kinases1 / 2 (ERK1/2) activation in ischemic kidney [84]. The TR diet reduces damage to epithelial cell of proximal tubule, death of tubular cell and activation of ERK1/2 in response to stress [30]. The CR improves AKI by increasing autophagy and dealing with reduced renal expression of endothelial nitric oxide synthase (eNOS), and PGC-1a caused by kidney damage. It also reduces acute tubular necrosis, and prevents reduction of renal function during injury [85]. But more research is needed in the future to discover the molecular mechanisms of this effects.

We only used male animals in this study, which could be a potential limitation. Nevertheless, the research methodology was designed to minimize gender dependency. In our next study, the tests will be performed on female animals with estrous cycle synchronization or on ovariectomized animals.

## Conclusions

Findings of this study showed that regular moderate-intensity exercise before induction of AKI reduces injury, inflammation and oxidative stress following AKI and also leads to less reduction in SIRT1 in those with previous exercise compared to those without exercise. When the two CR and TR regimens are applied, the renoprotective effects of exercise are greater in AKI, and among these two regimens, the effect of TR regimen is greater.

## Data Availability

All data generated or analyzed during this study are included in this published article.

## Abbreviations

AGE: Advanced glycation end products
AKI: Acute Kidney Injury AP-1:Protein Aactivator-1
CKD: Chronic Kidney Disease
CR: Calorie Restriction
ERK1 / 2: Extracellular signal-Regulated Kinases1 / 2
ESKD: End-Stage Kidney Disease
FRAP: Ferric reducing antioxidant power
GFR: Glomerular Filtration Rate
GPx: Glutathione Peroxidase
IL-1: Interleukin-1
IL-6: Interleukin-6
IL-18: Interleukin-18
IR: Ischemia-reperfusion
eNos: endothelial Nitric Oxide Syntheses
MAPK: Mitogen- Activated Protein Kinase
MDA: Malondialdehyde
NF-κB: Nuclear Factor kappa B
NO: Nitric oxide
NRF2: Nuclear factor-erythroid factor 2-related factor 2
PGC1α: Peroxisome proliferator-activated receptor Gamma Co-activator 1-α
SIRT1: Silent information regulator1
SOD: Superoxide dismutase
TAC: Total antioxidant capacity
TBA: Thiobarbituric acid
TGF-β: Transforming Growth Factor-β
TNF-α: Tumor Necrosis Factor-α
TPTZ: 2, 4, 6-tri-pyridyl-s-3, 6 triazine
TR: Time restriction
